# Secular trends in HIV knowledge and attitudes among Vietnamese women based on the Multiple Indicator Cluster Surveys, 2000, 2006, and 2011: what do we know and what should we do to protect them?

**DOI:** 10.3402/gha.v9.29247

**Published:** 2016-02-29

**Authors:** Nguyen Van Huy, Hwa-Young Lee, You-Seon Nam, Nguyen Van Tien, Tran Thi Giang Huong, Luu Ngoc Hoat

**Affiliations:** 1 Department of Health Management and Organization, Institute for Preventive Medicine and Public Health, Hanoi Medical University, Hanoi, Vietnam; 2 JW Lee Center for Global Medicine, College of Medicine, Seoul National University, Seoul, Korea; 3 Department of Family Medicine, Seoul National University Hospital, Seoul, Korea; 4 Department of Health Management and Organization, Faculty of Public Health, Thaibinh Medical University, Hanoi, Vietnam; 5 Department of Policy and Integration, Hanoi School of Public Health, Hanoi, Vietnam; 6 Department of Biomedical Statistics and Informatics, Institute for Preventive Medicine and Public Health, Hanoi Medical University, Hanoi, Vietnam

**Keywords:** HIV/AIDS knowledge, HIV/AIDS attitude, MICS, Vietnamese women, Vietnam

## Abstract

**Background:**

In Vietnam, women are at risk of HIV infection due to many factors. However, there is limited evidence about what women know and how they behave to protect themselves from HIV.

**Objective:**

The objective of this study was to investigate the trends in comprehensive HIV/AIDS knowledge, attitude, and associated factors among Vietnamese women from 2000 to 2011.

**Design:**

Data from three waves of the Vietnam Multiple Indicator Cluster Surveys (years 2000, 2006, and 2011) were used. Logistic regression methods examined factors associated with each of two dependent variables, HIV/AIDS knowledge and attitude toward HIV/AIDS.

**Results:**

Although there was an increasing trend in basic HIV/AIDS knowledge and positive attitude toward the disease, in Vietnamese women in the general population over the survey years, the prevalence of women with basic HIV/AIDS knowledge and positive attitude toward HIV/AIDS was low. Multivariable models indicated that women who had higher levels of education, lived in urban areas, had higher economic status, and knew about places of HIV-related services were more likely to have good HIV/AIDS knowledge (e.g. in 2011, AOR's=3.01; 1.27; 1.88; 2.03, respectively). Women with higher educational attainment, knew about HIV services, and had better HIV knowledge were more likely to report positive attitude toward HIV/AIDS (e.g. in 2011, AOR's=2.50; 1.72; 2.23, respectively).

**Conclusions:**

This study recommends that public health programs for the control of HIV, such as behavioral change communication campaigns or social policies for women, should focus not only in improving the quality of existing HIV/AIDS counseling and testing services but also on expanding coverage to increase accessibility to these services for women in rural areas. In addition, efforts to raise the level of knowledge about HIV/AIDS and improve attitude toward the disease should be undertaken simultaneously. The results of this study can help inform HIV control policies and practices in other developing countries.

## Introduction

The HIV epidemic in Vietnam is still at a ‘concentrated stage’ according to the World Health Organization (WHO) and the Joint United Nations Programme on HIV/AIDS (UNAIDS) classification (1). This means that HIV infection in Vietnam is high among the most-at-risk population, although the HIV epidemic remains less than 1% in the general population. Of concern is the fact that the prevalence of HIV in high-risk groups is increasing (2). Consequently, the Vietnamese government's efforts have focused mainly on recognized high-risk groups.

**Fig. 1:**
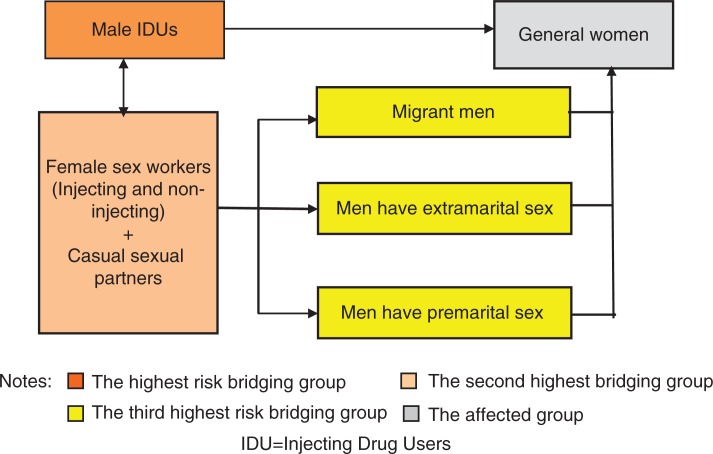
Possible routes of HIV transmission in Vietnam (3).

Given the current context of the HIV epidemic in Vietnam, in which there are complex interactions within the transmission mechanisms, women, as well as those in high-risk groups, are vulnerable to HIV infection. There are numerous high-risk ‘bridging groups’ through which HIV can be transmitted to women (see Fig. 1). Male injecting drug users (IDUs) represent the biggest threat to women in the general population in terms of behavioral characteristics and lifestyle because these men usually share injecting equipment and do not use a condom during sexual relations (4). Migrant men and men who have pre or extra-marital sex are another high-risk group for women in the general population. Female sex workers and other casual sex partners indirectly transmit HIV to women in the general population through IDUs and encounters with men. Given these routes, it is of concern that the current epidemic of HIV in Vietnam could move to the general population. Evidence of this is already seen in Vietnamese provinces such as Hochiminh, Haiphong, and Quangninh, where over 1.1% of the general population is recorded as being HIV infected (5, 6).

In a wider social context, women in developing countries, such as Vietnam, are more vulnerable to HIV infection than men. In general, educational levels are lower among women, and there are social and cultural taboos associated with the discussion of topics related to sex (7). In addition, women often play a submissive role in sexual relationships and therefore can have very limited availability of, and accessibility to, HIV care and prevention services.

Until now, the Vietnamese government's efforts to prevent the spread of HIV have focused on recognized high-risk groups such as young male IDUs (3). However, given the potential vulnerability of women to HIV infection, it is high time to expand healthcare professionals’ and policy makers’ attention to women in the general population. Previous studies have identified HIV/AIDS knowledge and HIV risk perception and attitude as important influences on the low utilization of HIV control programs (8–10). Hence, greater understanding of factors that can influence women's knowledge and attitude toward the disease will be important in preventing further spread of HIV.

Although previous studies about the determinants of HIV/AIDS knowledge and attitude are not rare, they have tended to target specific groups such as married women (11), healthcare professionals (12), migrant males (13), or residents in specific local geographic areas within a country. For example, Kobeissi et al. (11) assessed the association between married women's knowledge of HIV/AIDS transmission and prevention and socioeconomic status using 2004 survey data in Lebanon. The authors reported that region, level of income, and level of education were significantly associated with HIV/AIDS knowledge. Navaratna et al. (14) investigated possible associations between HIV-related variables and socioeconomic indicators in the general population of Kandy, Sri Lanka. Their findings showed a significant positive correlation between positive attitude toward people living with HIV/AIDS and HIV/AIDS knowledge, but no significant associations with other sociodemographic variables were found.

Many previous studies of HIV/AIDS knowledge and attitude have targeted African countries because sub-Saharan Africa has had the highest prevalence of HIV in the world. Gurmu et al. (15) examined factors associated with the stigmatization of people living with HIV in Ethiopia and showed that women with higher levels of education and adequate knowledge about HIV/AIDS were less likely to be stigmatized. Burgoyne and Drummon (16) investigated the current state of knowledge of HIV/AIDS in women in sub-Saharan Africa. Their study included a snapshot whereby those with the least knowledge were poorly educated women, especially those from rural backgrounds, and women who were economically dependent on men. Ochako et al. (17) investigated the main correlates of HIV/AIDS knowledge among young Kenyan urban women and identified four factors (education, experience of HIV testing, knowing someone infected with HIV, and having risk perception) as important.

On the other hand, an increasing number of studies have targeted Asian countries in recent years. Atteraya et al. (18) explored ethnicity and caste-based inequality in HIV-related knowledge among women in Nepal and found a significant association. Tang et al. (19) addressed questions about different patterns of sexual attitude and levels of sex-related knowledge among unmarried female migrant workers in China. Based on the results that unmarried migrant female workers lacked sexual knowledge and a substantial proportion were engaged in premarital sexual behaviors; this study recommended that effective interventions, aimed at improving sexual knowledge and related skills, were needed. To our knowledge, no studies have investigated the determinants of HIV/AIDS knowledge or attitude among Vietnamese women in the general population. One related study conducted in Thai Nguyen, Vietnam, investigated the individual- and community-level determinants of stigma toward people living with HIV who inject drugs (18). The results showed that socioeconomic factors operating at both the individual and community level were important. However, the findings do not provide sufficient evidence for understanding broader attitude toward HIV/AIDS in women in the general population in Vietnam.

In order to develop appropriate intervention strategies for women, it is essential to assemble evidence of the current state of HIV/AIDS knowledge, attitude, and associated factors. The objective of this study was to investigate factors associated with HIV/AIDS knowledge and attitude toward HIV/AIDS among Vietnamese women. The purpose is to provide evidence to contribute to the development of future programs for the prevention and management of HIV/AIDS.

## Methods

### Data source

All the variables were derived from the Multiple Indicator Cluster Surveys (MICS) in Vietnam (20–22), which were conducted on a large scale by the General Statistics Office in collaboration with the Ministry of Health (MOH) and the Ministry of Labour, Invalids and Social Affairs (MOLISA). Financial and technical support for the surveys came from the United Nations Children's Fund (UNICEF) and the United Nations Population Fund (UNFPA). The MICS are repeated cross-sectional surveys, which target different respondents each year. They generate national representative data covering a broad range of issues affecting the health, development, and living conditions of Vietnamese women and children. Three waves of data collection, undertaken in 2000, 2006, and 2011, are currently available. The MICS datasets and results are publicly accessible via a website.^1^

Each survey wave is composed of three well-structured separate datasets giving information on households, women aged 15 – 49 years, and children below the age of 5 living in Vietnam. All the variables for this study were drawn from the women's datasets in 2000 (*N*=9,295), 2006 (*N*=9,471), and 2011 (*N*=12,115). We restricted our sample for the final analysis to those who had a valid response in all of the outcome and independent variables. After removing records with missing data, the numbers of women in the final analysis were 9,117, 9,471, and 11,614 from the surveys in 2000 (21), 2006 (20), and 2011 (22), respectively.

### Measures

#### Dependent variables

*Basic knowledge of HIV/AIDS* was measured using five ‘yes/no/don't know’ items as in Table 2. Knowledge scale scoring was measured by dichotomizing each item into a value of 1 (correct) and 0 (incorrect or don't know) and then summing the item values to a composite score with higher scores reflecting increased knowledge about HIV prevention. Respondents were assessed as having good basic knowledge of HIV/AIDS if they correctly answered all five questions, i.e. when the composite score was 5, and as having incomplete basic knowledge if they gave a wrong answer on any one of the five questions, i.e. when the composite score was lower than 5.

*Attitude toward HIV/AIDS* were also measured using two ‘yes/no/don't know’ items as shown in Table 3. The attitude scale scores were derived by dichotomizing each item into a value of 1 (yes=positive attitude) and 0 (no or don't know=negative attitude) and then summing the item values to a composite score with higher scores reflecting better attitude toward HIV/AIDS. Respondents who had a composite score of 2, meaning that they positively accepted both situational questions, i.e. willingness to accept a teacher infected with HIV to teach at school and willingness to buy fresh vegetables from a shopkeeper or vendor infected with HIV, were categorized as having-positive-attitude group. Respondents who had a composite score of less than 2 were categorized as having-negative-attitude group.

#### Predictor variables

Independent variables were the factors that previous studies had shown to be associated with the outcomes. *Age* was dichotomized into two groups: <30 years and ≥30 years, the reference group including the mean age of the study population (23). *Marital status* was categorized as being single (including ‘widowed/divorced/separated’ and ‘never married status’) and currently married (23). *Ethnicity* was assessed by asking if respondents were from the *Kinh*, the largest ethnic group in Vietnam, or from another (minority) ethnic group (17). *Educational levels* were classified as having completed primary, lower, or higher secondary school (17). *Living area* was assessed by asking if respondents were from urban or rural areas (23). Economic status was assessed differently between rural (mostly by assets) and urban areas (mostly by income and/or employment status). As the majority of women in the MICS survey were from rural areas, a household wealth index was used as a proxy variable for *economic status*. This captured underlying long-term wealth based on household information about ownership of consumer goods, dwelling characteristics, and water and sanitation. Wealth scores were derived using principal component analysis. The scores were ranked and grouped into quintiles from the poorest to the wealthiest. *Knowing about places to get counseling and testing for HIV* was measured by directly asking if respondents knew of any places where they could get HIV/AIDS counseling and testing (24).

### Data analysis

First, descriptive statistics gave the overall picture of HIV/AIDS knowledge and attitude of Vietnamese women. Second, inferential analyses were undertaken using multivariable logistic regression. Two predictive models were examined separately for factors associated with 1) good basic knowledge of HIV/AIDS and 2) positive attitude toward HIV/AIDS. Independent variables that showed significant association with either of the outcome variables in the univariable analysis remained in a multivariable analysis. Final models were determined using indices of model fit (*p*-values of the model coefficients being <0.05 and the Hosmer–Lemeshow test statistic >0.05) (25).

## Results

### Selected characteristics of the sample

Selected sociodemographic characteristics of the study population in each of the three survey years, 2000, 2006, and 2011, are described in Table 1. The mean age of the women in the study sample was around 30, and most were married, of Kinh ethnicity, and living in rural areas. The proportion of women with upper secondary education increased from 23.4% in 2000 to 42.2% in 2011. Table 1 shows that Vietnamese women who were younger, single, of Kinh ethnicity, and from urban areas and had higher education and higher economic status knew about places to receive HIV/AIDS counseling and testing, reported good knowledge of HIV/AIDS, and displayed more positive attitude toward HIV/AIDS.

**Table 1:** Descriptive characteristics of representative samples of women in MICS, 2000, 2006, and 2011

	Proportion of observations			% of accurate knowledge			% of positive attitude		
			
Variables	2000 (*N*=9,252)	2006 (*N*=9,471)	2011 (*N*=11,663)	2000	2006	2011	2000	2006	2011
Age									
Range	29.9±9.9	30.9±10.5	31.47±9.9						
< 30	46.9	46.9	46.6	23.7	40.3	43.8	25.4	44.8	51.6
≥ 30	53.1	53.1	53.5	22.8	33.6	43.7	21.5	38.4	49.8
Marital status									
Single^a^	35.8	34.5	29.8	25.2	44.0	52.2	27.7	48.4	59.6
Currently married^b^	64.1	65.6	70.3	22.9	32.9	44.0	21.2	37.6	51.8
Educational level									
Primary	35.2	40.1	21.4	8.6	28.9	21.1	9.02	32.2	28.7
Lower secondary	41.3	44.7	36.4	23.8	35.7	40.1	24.6	42.3	52.9
Upper secondary	23.4	15.3	42.2	46.3	60.5	64.6	43.4	62.5	68.1
Ethnicity									
Ethnic minority	21.3	22.9	15.7	11.2	26.7	28.6	11.4	25.6	34.9
Kinh	78.7	77.1	84.3	27.1	39.7	49.7	26.8	46.0	57.7
Economic status									
Poorest quintile	21.8	21.3	18.5	7.3	21.7	27.2	8.45	21.4	35.8
Second quintile	20.3	18.2	16.5	15.5	30.2	36.8	17.9	36.3	48.1
Middle quintile	19.4	19.2	19.1	24.4	31.4	41.9	26.3	40.6	55.3
Fourth quintile	18.5	19.9	21.7	31.2	41.9	50.3	32.4	50.4	60.6
Richest quintile	20.1	21.5	24.3	42.4	57.3	67.4	34.6	57.8	65.4
Living area									
Rural	74.8	74.9	55.6	18.3	31.0	37.7	20.1	36.7	48.9
Urban	25.2	25.1	44.4	39.9	53.9	57.2	33.5	55.3	60.6

a Respondents were widowed, divorced, separated, or never married status

b respondents were currently married or living with their partners at the time of MICS survey.

### HIV/AIDS knowledge and attitude among Vietnamese women

As shown in Table 2, the percentages of women with comprehensive basic knowledge about HIV prevention were low in all three rounds of the MICS. For example, about 30 – 50% of women wrongly believed that people can be infected with HIV from mosquito bites. About 20% to almost 30% of women believed that you can tell by the appearance of someone if they have HIV or not. In 2006 and 2011, about 90% of women correctly reported that having just one uninfected sex partner who has had no other sex partners, or always using a condom when having sex, can reduce the chances of contracting HIV. More than 90% did not believe that people can get HIV because of witchcraft or other supernatural means. The proportion of women who correctly responded to all five items about HIV transmission was 23.7, 36.4, and 44.5% in 2000, 2006, and 2011, respectively.

**Table 2:** Percentage of correct responses to comprehensive basic knowledge of HIV/AIDS of representative samples of women in MICS, 2000, 2006, and 2011

	% of correct responses		
Questions	2000	2006	2011
1. People can reduce their chance of getting HIV by having just one uninfected sex partner who has no other sex partners	81.5	90.2	89.6
2. People can get HIV because of witchcraft or other supernatural means	94.4	96.2	92.9
3. People can reduce their chance of contracting HIV by using a condom every time they have sex	84.2	94.2	90.0
4. People can get HIV from mosquito bites	53.8	63.2	69.5
5. It is possible for a healthy-looking person to have HIV	69.6	84.4	76.6
6. Correctly identify all 5 misconceptions about HIV transmission	23.7	36.4	44.7

Table 3 shows that there were relatively low proportions of women with positive attitude toward HIV/AIDS in all three rounds of the MICSs. In 2000, only 56.1% said that a female teacher infected (but not sick) with HIV should be allowed to continue teaching in school, while this percentage increased to 63.4% in 2006 and 70% in 2011. Similarly, in 2000, just over 30% reported that they would buy fresh vegetables from a shopkeeper or vendor if they knew that this person had HIV, while this proportion increased to 55.4 and 64.8% in 2006 and 2011, respectively. Although the proportions of Vietnamese women with positive attitude to both of these issues improved across the survey rounds, they remained low at 23.5, 41.4, and 52.1% in 2000, 2006, and 2011, respectively.

**Table 3:** Percentage of positive attitude toward HIV/AIDS of representative samples of women in MICS, 2000, 2006, and 2011

	% of positive attitude		
Questions	2000	2006	2011
1. In your opinion, if a female teacher has HIV but is not sick, should she be allowed to continue teaching in school?	56.1	63.4	70.0
2. Would you buy fresh vegetables from a shopkeeper or vendor if you knew that this person had HIV?	31.9	55.4	64.8
Positively accept all 2 attitude toward people living with HIV	23.5	41.4	52.1

### Factors associated with HIV/AIDS knowledge and attitude among Vietnamese women

In the multivariable analysis (Table 4), four factors (higher educational level, urban living area, higher economic status, and knowledge of places for counseling and testing) were significantly associated with *good basic knowledge of HIV/AIDS* in both 2000 and 2011. Specifically, women who attained higher level of education, were living in rural areas, had higher economic status, and knew about the places for HIV/AIDS services, showed significantly higher odds of having good basic knowledge of HIV/AIDS. In the 2006 survey, all seven independent variables were significantly associated with good knowledge of HIV/AIDS. All three models were a good fit of the data (*p*-value of the model coefficients <0.05 and Hosmer–Lemeshow test >0.05).

**Table 4:** Multivariable logistic regression of factors associated with HIV/AIDS knowledge among women in MICS, 2000, 2006, and 2011

	AOR (95% CI)		
	
Predictors	2000 (*N*=9,117)	2006 (*N*=9,471)	2011 (*N*=11,614)
Age group			
< 30	1	1	1
≥ 30	1.01 (0.88–1.15)	*0.79 (0.69–0.89)	0.81 (0.55–1.17)
Marital status			
Single	1	1	1
Married	0.98 (0.85–1.12)	**0.73 (0.64–0.83)	0.91 (0.69–1.19)
Ethnicity			
Ethnic minority	1	1	1
Kinh	1.12 (0.91–1.38)	*0.81 (0.59–0.85)	0.99 (0.80–1.24)
Educational levels			
Primary	1	1	1
Lower secondary school	*1.69 (1.42–2.01)	*1.13 (1.01–1.27)	*1.88 (1.58–2.24)
Upper secondary school	*3.56 (2.95–4.29)	*2.02 (1.71–2.37)	*3.01 (2.46–3.69)
Living area			
Rural	1	1	1
Urban	*1.30 (1.11–1.52)	*1.31 (1.13–1.51)	*1.27 (1.09–1.48)
Economic status			
Poorest quintile	1	1	1
Second quintile	*1.44 (1.11–1.88)	1.10 (0.89–1.34)	1.00 (0.79–1.26)
Middle quintile	*1.97 (1.52–2.55)	1.08 (0.88–1.33)	1.05 (0.83–1.31)
Fourth quintile	*2.22 (1.71–2.87)	*1.53 (1.24–1.88)	*1.27 (1.00–1.61)
Richest quintile	*2.24 (1.69–2.95)	*2.33 (1.86–2.94)	*1.88 (1.44–2.47)
Knowing places to receive HIV/AIDS counseling and testing			
Did not know	1	1	1
Knew	*1.91 (1.67–2.18)	*2.35 (2.07–2.65)	*2.03 (1.76–2.33)
*p*-value of model coefficients	***	***	***
*p*-value (*χ* ^2^(*df*) of Hosmer–Lemeshow)	NS	NS	NS
Nagelkerke's *R* ^2^	10.96%	8.71%	9.10%

CI=confidence interval; AOR = adjusted odds ratio.

* *p*<0.05

** *p*<0.01

*** *p*<0.001.

Values in bold represent significant OR.

Table 5 shows factors associated with positive attitude toward HIV/AIDS. All independent variables were statistically significant, except for age and living area in 2000 and ethnicity and living area in 2006. In 2011, only three factors were significantly associated with positive attitude toward HIV/AIDS – educational level, knowledge of places for counseling and testing, and HIV/AIDS basic knowledge. In 2011, women who reported having reached lower secondary school and upper secondary school had higher odds of having positive attitude toward HIV/AIDS (OR 1.78; CI: 1.51–2.09) and (OR 2.50; CI: 2.05–3.05), respectively.

**Table 5:** Multivariable logistic regression of factors associated with HIV/AIDS attitude among women in MICS, 2000, 2006 and 2011

	AOR (95% CI)		
	
Predictors	2000 (*N*=9,117)	2006 (*N*=9,471)	2011 (*N*=11,814)
Age group			
< 30	1	1	1
≥30	0.91 (0.79–1.04)	**0.83 (0.73–0.94)**	0.98 (0.67–1.44)
Marital status			
Single	1	1	1
Married	**0.79 (0.69–0.91)**	**0.79 (0.69–0.91)**	0.94 (0.71–1.24)
Ethnicity			
Ethnic minority	1	1	1
Kinh	**1.39 (1.12–1.72)**	0.90 (0.75–1.08)	1.14 (0.92–1.40)
Educational levels			
Primary	1	1	1
Lower secondary school	**1.53 (1.29–1.82)**	**1.19 (1.06–1.34)**	**1.78 (1.51–2.09)**
Upper secondary school	**2.71 (2.23–3.30)**	**1.57 (1.33–1.86)**	**2.50 (2.05–3.05)**
Living area			
Rural	1	1	1
Urban	0.89 (0.76–1.06)	1.01 (0.87–1.18)	0.98 (0.84–1.13)
Economic status			
Poorest quintile	1	1	1
Second quintile	**1.58 (1.23–2.22)**	**1.24 (1.01–1.52)**	1.07 (0.86–1.32)
Middle quintile	**1.92 (1.51–2.44)**	**1.36 (1.10–1.63)**	1.15 (0.92–1.43)
Fourth quintile	**2.11 (1.65–2.69)**	**1.70 (1.37–2.11)**	1.14 (0.91–1.44)
Richest quintile	**1.58 (1.21–2.09)**	**1.84 (1.44–2.34)**	1.09 (0.83–1.42)
Knowing places to receive HIV/AIDS counseling and testing			
Did not know	1	1	1
Knew	**1.71 (1.50–1.96)**	**2.22 (1.96–2.51)**	**1.72 (1.50–1.96)**
Comprehensive basic knowledge of HIV/AIDS			
Lower	1	1	1
Higher	**2.33 (2.05–2.64)**	**2.49 (2.23–2.78)**	**2.23 (1.94–2.55)**
*p*-value of model coefficients	***	***	***
*p*-value (*χ* ^2^(*df*) of Hosmer–Lemeshow)	NS	NS	NS
Nagelkerke's *R* ^2^	10.64%	10.34%	8.49%

CI=confidence interval; AOR=adjusted odds ratio.

*** *p*<0.001.

Values in bold represent significant OR.

In 2011, women who knew about places to receive HIV/AIDS service were 70% more likely to have positive attitude toward HIV/AIDS (OR: 1.72; CI: 1.50–1.96). The odds of having a positive attitude toward HIV/AIDS were more than twice as high in women who had comprehensive basic knowledge of HIV/AIDS in each of the three survey years (AOR: 2.33 in 2000, 2.49 in 2006, and 2.23 in 2011). All three models were a good fit to the data (*p*-value of the model coefficients <0.05 and Hosmer–Lemeshow test >0.05).

## Discussion

This study investigates the trends and determinants of comprehensive HIV/AIDS knowledge and positive attitude toward HIV/AIDS among Vietnamese women using national survey data collected in 2000, 2006, and 2011. Although there was a consistent increase in the proportion of women who had comprehensive knowledge of HIV/AIDS, the results in the latest survey year, 2011, were far below the 95% target proposed by the United Nations General Assembly Special Session (UNGASS) (26). A high proportion of women had misconceptions about the transmission routes and prevention methods of HIV. In particular, there was poor knowledge about the transmission route due to mosquito bites. People need to know not only about the disease itself but also about transmission methods in order to protect themselves from infection. Therefore, lack of correct informed knowledge will put Vietnamese women at greater risk of HIV infection.

The results of the trend in positive attitude toward HIV/AIDS showed a similar pattern. The proportion of women with positive attitude almost doubled between 2000 and 2011. This may be in part due to the increased proportion of women with comprehensive knowledge about HIV/AIDS because more accurate knowledge can lead to more positive attitude toward HIV/AIDS. Nevertheless, the percentage of women with positive attitude barely managed to exceed 50% in 2011, which could mean that people with HIV in Vietnam are stigmatized. In such a social climate, people having HIV tend to hide their disease and do not seek proper services such as screening tests or treatments for fear that their disease status would be made known to others, which in turn, promotes the diffusion of the disease (27, 28). In addition, discriminatory behavior against people living with HIV often violates patients’ human rights (29).

Some significant findings arose from the analysis of factors associated with inadequate and incomprehensive knowledge of and attitude toward HIV/AIDS among Vietnamese women. First, given the change in significance of each variable across the three time points, we can assume that some groups are more receptive to new HIV/AIDS knowledge than others. For example, women were generally ignorant about HIV/AIDS in 2000, with no significant differences between age groups. However, in 2006, women above 30 years old showed significantly lower odds of having accurate knowledge compared with women aged below 30 years. However, this difference between age group was not seen in 2011, suggesting that knowledge about HIV/AIDS may spread among younger women faster, but older women may have improved their knowledge about HIV/AIDS by 2011. Younger women may be more interested in this information because they are more likely to be single and engage in sexual activities with different partners, and also because they are more adaptive to absorbing new information. The results by marital status also showed a similar pattern. The difference in the level of comprehensive knowledge of HIV/AIDS between single and married women was not significant in 2000. However, in 2006 married women had almost 30% lower odds of having comprehensive knowledge of HIV/AIDS compared with single women. One possible reason for this is that married women often believe that marriage is protective against HIV infection because marriage provides checks and balances on individuals’ sexual behavior, and that they will benefit from their husbands’ knowledge of HIV/AIDS (17, 30).

Second, associations between education level, economic status, and residential areas and knowledge of HIV/AIDS were significant in all three survey years. Specifically, women with higher education, higher economic levels, and those living in urban areas were more likely to have an accurate knowledge of HIV/AIDS. These results are consistent with the results of previous studies (31, 32). Higher education can lead to employment, higher income, and better access to information. Higher education may influence HIV/AIDS knowledge by providing women not only with the ability to get information on prevention, but also by encouraging them to take charge of their health as a future investment (33). Possible reasons why women residing in rural areas were less likely to be knowledgeable about HIV/AIDS may be that HIV counseling and testing (HCT) services were not only less available to people in rural areas, but also running HIV/AIDS-related campaigns in remote areas is more difficult. Therefore women in rural areas are less likely to be exposed to information about HIV/AIDS. In these 3 years, women in the highest economic quintile were significantly more knowledgeable than women in the lowest economic quintile.

A notable finding is that women who were aware of where to get HCT were about twice as likely to be knowledgeable about HIV/AIDS compared with those who were not aware of places to receive HCT. People can access information about HIV/AIDS and even arrange tests for their HIV serostatus through the HCT services. This highlights the importance of ensuring that HCT services are widely available and accessible.

Lastly, significant associations remained even after controlling for the level of knowledge of HIV/AIDS. This suggests that there may be other mechanisms affecting positive attitude toward HIV/AIDS in addition to basic HIV/AIDS knowledge. There is a need for further investigation of these issues, possibly using qualitative methods such as in-depth interviews.

To our knowledge, this is the first study of its kind to identify factors related to accurate HIV/AIDS knowledge and positive attitude toward HIV/AIDS in Vietnamese women. However, there are some limitations. First, although there are more items asking about knowledge and attitude of HIV/AIDS in the MICS questionnaire, we did not use all of these because they were not consistently surveyed in these three periods. Another limitation is that some variables of interest were not available in the MICS dataset. It is therefore possible that some important factors were omitted.

Considering the gap between the current level of knowledge and positive attitude among Vietnamese women and the target level set out by UNGASS, it is still a long way to go. However, from a policy perspective, the results of this study can be seen as a starting point. Firstly, this study emphasized a vulnerable group in the general population to which policy makers need to pay attention. Specifically, concerted efforts for raising the level of knowledge about HIV/AIDS should be directed toward women who are deprived in terms of education and economic status and accessibility to HIV/AIDS-related service, which is critical. However, there is, for example, a scarcity of antenatal care centers and maternity hospitals that provide routine and reliable voluntary counseling and testing in Vietnam (34). In addition, majority of these services are provided in urban areas, making it difficult for women in rural areas to access services (34). Efforts to improve the level of knowledge about HIV/AIDS should include not only improvements in the quality of the pre-existing services but also expansion in the coverage of existing services.

Another notable implication from the results of this study is that we should understand that separately tackling either knowledge or attitude alone through education is not sufficient. The two are closely interlinked. Accurate knowledge can protect people from contracting HIV, while positive attitude toward HIV can encourage those already affected to seek the necessary prevention and treatment without concealment. Having inaccurate knowledge about the transmission and prevention of HIV can cause fear leading to stigma and discrimination. One consequence of this is that HIV/AIDS patients may refuse to seek appropriate prevention, treatment, and care services, even if they have some knowledge of HIV/AIDS. Therefore, comprehensive measures for both the diffusion of HIV/AIDS knowledge and openness regarding HIV/AIDS attitude are needed.

## Conclusions

The results of this study will contribute to the understanding of HIV/AIDS knowledge and attitude among Vietnamese women. It is important to expand AIDS control policies, not only among high-risk groups but also among Vietnamese women who are vulnerable to HIV infection. As Vietnam has much in common with other developing countries in Southeast Asia, this study could also inform policy makers and practitioners in other developing countries. The time to act is now.

## Footnotes

1 mics.unicef.org

## References

[1] UNAIDS (2006). Monitoring and evaluation of HIV prevention programmes for most-at-risk populations. A framework for monitoring and evaluation HIV prevention programmes for most-at-risk populations.

[2] Socialist Republic of Vietnam (2005). Second country report on following up the implementation to the declaration of commitment on HIV/AIDS January 2003 – December 2005. http://www.unaids.org.vn/resource/topic/natstrat/ungass_17jan06_e.pdf.

[3] Vietnam Ministry of Health (2004). HIV/AIDS estimates and projections 2005–2010. http://unaidsorgvn/resource/topic/epidemiology/e&p_english_finalpdf.

[4] Huy NV A potential HIV epidemic among male street laborers in urban Vietnam.

[5] UN Vietnam Official HIV/AIDS estimates and projections for Vietnam 2005–2010 and Vietnam's 2nd UNGASS report 2006. http://www.unaids.org.vn/facts/docs/key_messages_sep_2006_e.pdf.

[6] Vietnam Ministry of Health, Health Partnership Group (2008). Health status and determinants of health (Chapter 1).

[7] World Health Organization (2003). HIV infected women and their families: psychosocial support and related issues.

[8] Boulle A, Hilderbrand K, Menten J, Coetzee D, Ford N, Matthys F (2008). Exploring HIV risk perception and behaviour in the context of antiretroviral treatment: results from a township household survey. AIDS Care.

[9] Haile BJ, Chamber JW, Garrison JL (2007). Correlates of HIV knowledge and testing: results of a 2003 South African HIV Survey. J Black Stud.

[10] Bazargan M, Kelly EM, Stein JA, Husaini BA, Bazargan SH (2000). Correlates of HIV risk-taking behaviors among African-American college students: the effect of HIV knowledge, motivation, and behavioral skills. J Natl Med Assoc.

[11] Kobeissi L, El Kak FH, Khawaja M, Khoshnood K (2015). HIV/AIDS-related knowledge and its association with socioeconomic status among women: results of Lebanese Survey for Family health (PAPFAM) 2004. Asia Pacific J Public Health.

[12] Memish ZA, Filemban SM, Bamgboyel A, Al Hakeem RF, Elrashied SM, Al-Tawfiq JA (2015). Knowledge and attitudes of doctors toward people living with HIV/AIDS in Saudi Arabia. J Acquir Immune Defic Syndr.

[13] Yang B, Wu Z, Schimmele CM, Li S (2015). HIV knowledge among male labor migrants in China. BMC Public Health.

[14] Navaratna S, Kanda K, Dharmaratne SD, Tennakoon S, Jayasinghe A, Jayasekara N (2015). Awareness and attitudes towards HIV/AIDS among residents of Kandy, Sri Lanka. AIDS Care.

[15] Gurmu E, Etana D (2015). HIV/AIDS knowledge and stigma among women of reproductive age in Ethiopia. Afr J AIDS Res.

[16] Burgoyne AD, Drummond PD (2009). Knowledge of HIV and AIDS in women in sub-Saharan Africa. Afr J Reprod Health.

[17] Ochako R, Ulwodi D, Njagi P, Kimetu S, Onyango A (2011). Trends and determinants of comprehensive HIV and AIDS knowledge among urban young women in Kenya. AIDS Res Ther.

[18] Atteraya M, Kimm H, Song IH (2015). Caste- and ethnicity-based inequalities in HIV/AIDS-related knowledge gap: a case of Nepal. Health Soc Work.

[19] Tang J, Gao X, Yu Y, Ahmed NI, Zhu H, Wang J (2011). Sexual knowledge, attitudes and behaviors among unmarried migrant female workers in China: a comparative analysis. BMC Public Health.

[20] General Statistics Office, UNICEF (2007). Multiple indicator cluster survey 2006.

[21] General Statistics Office (2000). Multiple indicator cluster survey 2000.

[22] General Statistics Office (2011). Vietnam multiple indicator cluster survey 2011.

[23] Ndubuka J, Ndubuka N, Li Y, Marshall CM, Ehiri J (2013). Knowledge, attitudes and practices regarding infant feeding among HIV-infected pregnant women in Gaborone, Botswana: a cross-sectional survey. BMJ Open.

[24] Peltzer K, Matseke G, Mzolo T, Majaja M (2009). Determinants of knowledge of HIV status in South Africa: results from a population-based HIV survey. BMC Public Health.

[25] Hosmer DW, Lemeshow S (2000). Applied logistic regression.

[26] Joint United Nations Programme on HIV/AIDS, UNAIDS (2005). Monitoring the declaration of commitment on HIV/AIDS: guidelines on construction of core indicators.

[27] Lau JTF, Tsui HY (2003). Surveillance of discriminatory attitudes toward people living with HIV/AIDS among the general public in Hong Kong from 1994 to 2000. Disabil Rehabil.

[28] Peters L, den Boer DJ, Kok G, Schaalma HP (1994). Public reactions towards people with AIDS: an attributional analysis. Patient Educ Couns.

[29] UNAIDS (2005). HIV-related stigma, discimination and human rights violations: case studies of successful programmes.

[30] Akwara PA, Madise NJ, Andrew H (2003). Perception of risk of HIV/AIDS and sexual behaviour in Kenya. J Biosoc Sci.

[31] Central Statistical Agency (CSA) Ethiopia, ORC Macro (2006). Ethiopia demographic and health survey 2005.

[32] National Population Commission (NPC) Nigeria, ORC Macro (2004). Nigeria demographic and health survey 2003.

[33] Tenkorang EY, Rajulton F, Tyndale-Maticka E (2009). Perceived risks of HIV/AIDS and first sexual intercourse among youth in Cape Town, South Africa. AIDS Behav.

[34] Nguyen TA, Oosterhoff P, Hardon A, Tran HN, Coutinho RA, Wright P (2008). A hidden HIV epidemic among women in Vietnam. BMC Public Health.

